# High‐Risk Pregnancy Associated With Maternal Hypoparathyroidism and Medium‐Chain Acyl‐CoA Dehydrogenase Deficiency

**DOI:** 10.1155/crie/9924065

**Published:** 2026-06-04

**Authors:** Isabel Huguet, Cristina Sevillano Collantes, Laura Pérez-Olivares Martín, Guillermo Martínez Díaz-Guerra

**Affiliations:** ^1^ Department of Endocrinology, Hospital Infanta Leonor, Madrid, Spain; ^2^ Department of Endocrinology, Hospital 12 de Octubre, Madrid, Spain, madrid.org; ^3^ Department of Endocrinology, Instituto de Investigación Sanitaria i+12, Hospital 12 de Octubre, Universidad Complutense, Madrid, Spain, ucm.es

## Abstract

**Purpose:**

Fatty acid oxidation disorders are inherited metabolic errors that result in deficient energy production during prolonged fasting and catabolic stress. Few cases of hypoparathyroidism (HypoPT) related to medium‐chain acyl‐CoA dehydrogenase (MCAD) deficiency have been reported. We present the perinatal management of a female patient previously diagnosed with MCAD deficiency due to a homozygous G985A mutation and HypoPT.

**Case Presentation:**

A 24‐year‐old woman with MCAD deficiency and HypoPT presented with stage IV intrauterine growth restriction (IUGR) and preeclampsia at 28 weeks of gestation, requiring an emergency cesarean section. The fetus had been diagnosed with aortic arch hypoplasia and ventricular septal defect. Despite multidisciplinary management, the infant died at 8 months, with the underlying cardiopathy considered the most likely cause. This case highlights the challenges of managing coexisting metabolic and endocrine disorders during pregnancy.

**Conclusions:**

In this case, although MCAD deficiency may have contributed, HypoPT and its renal complications likely played a major role in adverse maternal and fetal outcomes. To the best of our knowledge, this is the first published case describing the coexistence of MCAD deficiency and HypoPT during pregnancy.

## 1. Introduction

Fatty acid oxidation disorders are inherited metabolic errors that result in deficient energy production during prolonged fasting and catabolic stress, and the unusual association of these disorders to hypoparathyroidism (HypoPT) has already been reported [1, 2]. Since transfer of carnitine from the mother to the fetus has been demonstrated during pregnancy [3–6], the carnitine supply during gestation could be of special interest in women affected by in medium‐chain acyl‐CoA dehydrogenase (MCAD) deficiency.

HypoPT during pregnancy must be frequently monitorized due to fluctuations in the requirements of treatment associated with the hormonal changes and the increase in intestinal absorption of calcium. Despite several case reports having described serious pregnancy‐specific complications such as excessive uterine contractions, the two main studies of chronic HypoPT in pregnancy [7, 8] reached heterogeneous conclusions.

While MCAD deficiency and HypoPT may individually be associated with adverse pregnancy outcomes, their coexistence has not been previously reported.

We report the case of a woman with HypoPT related to MCAD deficiency, with major complications for her and the fetus during pregnancy, and discuss the clinical course and obstetrical management.

## 2. Case Presentation

A 24‐year‐old woman was admitted to hospital for an elective caesarean at week 28 of gestation for intrauterine growth restriction (IUGR) stage IV and pathological cardiotocography.

Regarding her past medical history, at the age of 15 she was admitted with paresthesias and episodes of distal tetany. She was born to consanguineous parents of Eastern European Roma descent and had a prior diagnosis of MCAD deficiency due to a homozygous G985A mutation. Her brother was also affected, and the remainder of the family history was unremarkable. At that time, laboratory evaluation revealed severe hypocalcemia (6.7 mg/dL [reference range 8.5–10.6]), ionized calcium of 0.85 mmol/L [1.12–1.23], and inappropriately normal parathyroid hormone levels (19–28.1 pg/mL). She was diagnosed with primary HypoPT. No cardiomyopathy was identified, and given negative adrenal and parathyroid autoantibodies, normal magnesium levels, and absence of autoimmune disease or mucocutaneous candidiasis, HypoPT was considered likely related to MCAD deficiency.

At 20 years of age, she developed nephrocalcinosis and chronic kidney disease (estimated glomerular filtration rate 65–73 mL/min/1.73 m^2^) as complications of HypoPT.

At conception, her treatment included calcitriol 1.5 µg daily, calcium acetate 1 g every 8 h, and L‐carnitine 1 g every 8 h, with a serum calcium level of 9.3 mg/dL. Treatment was subsequently reduced to calcitriol 0.5 µg every 12 h and calcium acetate 1 g every 12 h. She declined preconception genetic counseling and had only been advised to discontinue hydrochlorothiazide.

Due to poor adherence to treatment and follow‐up, limited biochemical data were available during pregnancy. At 19 weeks of gestation, calcium was 9.6 mg/dL, phosphorus 3.4 mg/dL, magnesium 1.3 mg/dL, and estimated glomerular filtration rate 67 mL/min/1.73 m^2^.

At 19 weeks of pregnancy, the fetus was diagnosed with aortic arch hypoplasia and ventricular septal defect. At 25 weeks, increased resistance in the uterine artery and IUGR were diagnosed. The patient was not put on aspirin since she reported to be allergic to the drug. At 27 weeks she was diagnosed with pre‐eclampsia with increased levels of serum calcium and creatinine (11.9 and 1.61 mg/dL), increased levels of angiogenic biomarkers (soluble fms‐like tyrosine kinase 1 [sFlt‐1] 13,438.00 pg/mL, placental growth factor (PlGF) 17.9 pg/mL, ratio sFITt‐1/PlGF > 750) and was admitted to hospital for fetal lung maturation. At 28 weeks she gave birth to a son with a birth weight of 659 g, Apgar scores of 6/8/10, and pH 7.22–7.25.

Postpartum, maternal blood pressure normalized without further obstetric complications. Regarding the management of her HypoPT, the patient resumed follow‐up with calcium levels within the normal range.

Pathologic examination showed a small weight for gestation placenta <3rd centile, with accelerated maturation and >10% infarction as features of maternal vascular malperfusion.

Thrombophilia study was positive for lupus anticoagulants and negative for anticardiolipin antibodies. These results will have to be confirmed with a new test.

Unfortunately, the infant died at 8 months of age, with the underlying cardiopathy regarded as the most likely cause of this adverse outcome.

## 3. Discussion

Mitochondrial beta‐oxidation of saturated fatty acids is a metabolic pathway that satisfies energy requirements in humans, with MCAD deficiency being the most common enzymatic defect of these disorders. Although controversial, L‐carnitine supplementation has historically been used as part of long‐term management, as it may alleviate the secondary deficiency from acylcarnitine ester accumulation [9]. As several data suggest a transplacental transfer of carnitine from the maternal blood to the fetus in the middle stages of pregnancy [4, 5], carnitine status of pregnant women seems to be important for enabling mothers to supply adequately to the infant [3]. This supplementation could be of special interest in women affected by MCAD deficiency during gestation, and although scarce, some data suggest that carnitine deficiency could lead to premature preterm and low birth weight infants with intrauterine growth retardation [6].

HypoPT must be closely monitorized during pregnancy. While the study based on Swedish registry data for 139 pregnancies in women with HypoPT suggested a low risk of adverse outcomes [7], results from a population‐based study in the United States found significant increases in preterm birth and congenital anomalies [8]. Congenital anomalies in this study occurred despite adjusting for maternal age, obesity, chronic hypertension, thyroid disease, and pregestational diabetes. Unfortunately, data regarding the nature of these anomalies were unavailable, so the authors could not exclude genetic disorders associated with both chronic HypoPT and congenital anomalies with autosomal dominant heritance (DiGeorge syndrome, Kenny‐Caffey, and HypoPT Deafness Renal anomaly) Table 1. In our patient, poor treatment adherence and chronic renal complications may have contributed significantly to adverse outcomes.

**Table 1:** Comparison of major HypoPT‐in‐pregnancy Studies.

Study size (HypoPT group)	Björnsdottir et al. [7]	Hochberg et al. [8]
	*n*: 97	*n*: 204
Control group	1030 age‐matched controls.	9,096,584 pregnancies without HypoPT.
Primary etiology	76.4% postsurgical; 23.6% nonsurgical.	60% with a history of thyroid disease, suggesting postsurgical causes.
Preterm birth (<37 weeks)	Shorter gestational age, but not significant after adjusting for comorbidities (*p* = 0.119).	**Significant increase (19% vs. 7%, aOR: 2.49).**
Congenital anomalies	No difference compared to controls.	**Significant sixfold increase (4.4% vs. 0.4%, aOR: 6.50).**
Neonatal weight	Lower mean birth weight (−188 g) but no difference in small‐for‐gestational‐age.	Found comparable rates of small‐for‐gestational‐age.
Maternal complications	**Increased risk of induction of labor (aOR: 1.82).**	**Significant increase in blood transfusions (aOR: 4.07).**
Study conclusion	Majority of women have normal pregnancy outcomes; overall risk is low.	HypoPT increases the risk of adverse pregnancy, delivery, and neonatal outcomes.

*Note:* Bold text indicates statistical significance.

There are few descriptions in the literature of maternal fatty acid oxidation disorders and gestation outcomes. Some of the scarce cases have reported uneventfully completed gestation [10–13] and even some maternal cases of MCAD deficiency are diagnosed following the abnormal newborn screening of their infants [10, 11], having reached adulthood without severe symptoms or a life‐threatening decompensation. Interestingly, Santos et al. [14] described a maternal acute liver failure associated with an undiagnosed MCAD deficiency, with the delivery of a healthy boy in this context.

## 4. Conclusions

After reviewing the literature, although evidence remains inconclusive, we suggest that HypoPT and its renal complications were the main driver of the complications associated with pregnancy in our case. There is a need for further research on the interplay between HypoPT and fatty acid oxidation disorders in pregnancy.

## Funding

This work was supported by the Foundation for Biomedical Research and Innovation (FIIB) Infanta Leonor and Sureste Hospitals.

## Disclosure

All authors have read and approved the final version of the manuscript. Isabel Huguet had full access to all of the data in this study and takes complete responsibility for the integrity of the data and the accuracy of the data analysis.

## Consent

The authors confirm that they have the patient’s permission for her clinical information to be published in the journal and confirm patient understanding that clinical information will be published on an open access basis and may be freely accessed throughout the world.

## Conflicts of Interest

Isabel Huguet has participated as an advisor for Ascendis Pharma. Guillermo Martínez Díaz‐Guerra has participated as an advisor for Ascendis Pharma and Takeda. The others authors declare no conflicts of interest.

## Data Availability

The data that support the findings of this study are available upon request from the corresponding author. The data are not publicly available due to privacy or ethical restrictions.
